# CogNet: classification of gene expression data based on ranked active-subnetwork-oriented KEGG pathway enrichment analysis

**DOI:** 10.7717/peerj-cs.336

**Published:** 2021-02-22

**Authors:** Malik Yousef, Ege Ülgen, Osman Uğur Sezerman

**Affiliations:** 1 Galilee Digital Health Research Center (GDH), Zefat Academic College, Zefat, Israel; 2 Department of Information Systems, Zefat Academic College, Zefat, Israel; 3 Department of Biostatistics and Medical Informatics, School of Medicine, Acibadem Mehmet Ali Aydinlar University, Istanbul, Turkey

**Keywords:** Classification, Gene expression, Enrichment analysis, KEGG pathway, Rank, Machine learning, Bioinformatics, Data science, Data mining, Genomics

## Abstract

Most of the traditional gene selection approaches are borrowed from other fields such as statistics and computer science, However, they do not prioritize biologically relevant genes since the ultimate goal is to determine features that optimize model performance metrics not to build a biologically meaningful model. Therefore, there is an imminent need for new computational tools that integrate the biological knowledge about the data in the process of gene selection and machine learning. Integrative gene selection enables incorporation of biological domain knowledge from external biological resources. In this study, we propose a new computational approach named CogNet that is an integrative gene selection tool that exploits biological knowledge for grouping the genes for the computational modeling tasks of ranking and classification. In CogNet, the pathfindR serves as the biological grouping tool to allow the main algorithm to rank active-subnetwork-oriented KEGG pathway enrichment analysis results to build a biologically relevant model. CogNet provides a list of significant KEGG pathways that can classify the data with a very high accuracy. The list also provides the genes belonging to these pathways that are differentially expressed that are used as features in the classification problem. The list facilitates deep analysis and better interpretability of the role of KEGG pathways in classification of the data thus better establishing the biological relevance of these differentially expressed genes. Even though the main aim of our study is not to improve the accuracy of any existing tool, the performance of the CogNet outperforms a similar approach called maTE while obtaining similar performance compared to other similar tools including SVM-RCE. CogNet was tested on 13 gene expression datasets concerning a variety of diseases.

## Introduction

Due to recent advances in DNA gene expression technology, it is now feasible to obtain gene expression profiles of tissue samples at relatively low costs. Data from genome-wide gene expression analyses are helping scientists and physicians understand the disease mechanisms and use that information to design platforms to assist in diagnosis, to assess prognosis, and to inform treatment plans. For instance, a study by Van ’t Veer et al. (2002) collected gene expression data on primary breast tumors of 117 young patients. Machine learning with feature selection was used to identify a gene expression signature strongly predictive of a short interval to distant metastases (“poor prognosis” signature), even in patients that were lymph node negative.

Gene expression technologies are now producing large datasets associated with a variety of diseases. Due to the high dimensionality of the data and relatively small sample sizes, reliable interpretation of the data is a complicated and often overwhelming, and this is an important problem in bioinformatics research. Although sample sizes have continued to grow in recent years, new and efficient feature selection algorithms are still needed to overcome challenges in the existing methods (Vanjimalar, Ramyachitra & Manikandan, 2018), in order to achieve the full potential of this data in the development of gene-based diagnostic tests, drug discovery and therapeutic strategies for improving public health.

Most of the traditional gene selection approaches are borrowed from other fields such as statistics and computer science. There is a need for new computational tools that integrate the biological knowledge about the data in the process of gene selection and classification. Integrative gene selection incorporates biological domain knowledge to selection processes from external biological resources. The main aim of integrative gene selection is to generate a ranked list of features that provides high model performance and takes into consideration both statistical metrics applied on the gene expression data and the biological background information provided as external datasets. For example, biological background information may be Gene Ontology (GO) where it provides for each gene its product as Cellular Components (CC), Molecular Functions (MF), and Biological Processes (BP). GO is a way to capture biological knowledge in a computable form that consists from a set of concepts and their relationships with each other.

The various methods that have been applied to the process of selecting disease-specific features from large gene expression datasets were reviewed recently (Pan, 2002; Lazar et al., 2012) and fall into three major categories: “filters”, “wrappers”, and “embedded approaches”. Briefly, the filter approach, not based on any machine learning algorithm, uses a statistic (ANOVA, *t*-test, etc.), wrappers use learning techniques to evaluate which features are useful, and embedded techniques combine the feature selection step and classifier construction. Pan (2002) recently compared different filtering methods, highlighting similarities and differences between three main methods: the *t*-test, a regression modeling approach, and a mixture model approach. Additional comparisons of filtering techniques are available in Lazar et al. (2012). Inza et al. (2004) also carried out a comparison between a filter metrics and a wrapper sequential search procedure applied on gene expression datasets.

Integrative approaches become important topics (Bellazzi & Zupan, 2007; Fang, Mustapha & Sulaiman, 2014) in the emerging field of gene expression. GO (Ashburner et al., 2000) was used by Qi & Tang (2007) for genes ranking based on not only their individual discriminative powers but also the powers of biological information contained in GO annotations. The algorithm is an iterative algorithm that starts by applying Information Gain (IG) to compute discriminative scores for each gene. Genes with a score of zero are removed from the analysis. The second step is to integrate the biological knowledge by annotating those surviving genes with GO term. The third step is to score the GO terms as the mean of its associated gene IG score. Move the gene with the highest IG from the GO term with the highest score, to the final list. This procedure is repeated until the final goal is reached.

SoFoCles (Papachristoudis, Diplaris & Mitkas, 2010) is an interactive tool that enables semantic feature filtering in microarray classification problems with the use of external biological knowledge retrieved from the Gene Ontology. SoFoCles involves the calculation of semantic similarities between two feature sets in order to derive an enriched, semantically-aware final feature set. The GO terms are used in order to give a similarity score for each annotated gene.

Fang, Mustapha & Sulaiman (2014) proposed an integrative gene selection based on filter method and association analysis for selecting genes that are not only differentially expressed but also informative for classification. Association analysis was employed to integrate microarray data with Gene Ontology (GO) and KEGG Pathways (KEGG) simultaneously. The performance of the integrative models verified the efficiency and scalability of association analysis in mining microarray data.

An additional study that integrated KEGG with genetic meta information (DisGeNET (Piñero et al., 2019)) was proposed by Raghu et al. (2017). Their approach was a two-step analytical workflow that incorporates a new feature selection paradigm as the first step and that utilizes graphical causal modeling as the second step to handle the automatic extraction of causal relationships.

Quanz, Park & Huan (2008) apply the method of pathways-as-features using the KEGG pathway database for the pathway extraction component and global test method for the pathway selection component. The genes in each pathway are then transformed into one single feature by mean normalization or logistic regression. The number of features of the transformed data is the number of pathways. For instance, for the diabetes data for which 17 pathways are selected, the dimensionality is reduced from 22, 283 to 17 for the classification task.

Unsupervised gene selection using biological knowledge-based GO terms was suggested by Acharya, Saha & Nikhil (2017). They have utilized gene annotation data, where each gene is represented as a structural information content (IC) based gene-GO term annotation vector which intuitively forms a gene-GO term annotation matrix for a selected data set. IC is the information content of a GO term is related to how often the term is applied to genes in the database.

A very interesting study that emphasizes the need for an integrative approach was conducted by Perscheid, Grasnick & Uflacker (2019). Their work compared the performance of traditional and integrative gene selection approaches. Moreover, they propose a straightforward approach to integrate external knowledge with traditional gene selection approaches. The framework enables automatic external knowledge integration, gene selection, and evaluation. The study shows that the integration of external knowledge improves overall analysis results.

Feature selection and discovering the molecular explanation of disease describe the same process, where the first one is a computer science term and the second one is used in the biomedical sciences.

Several tools are now available that allow users to break the fixed set paradigm in assessing the statistical enrichment of sets of genes. In this regard, the gene set enrichment analysis is a very important method. Recently, different approaches were developed and become useful tools in gene expression analysis (Cohn-Alperovich et al., 2016; Ulgen, Ozisik & Sezerman, 2019). PathfindR (Ulgen, Ozisik & Sezerman, 2019) is a tool for pathway enrichment analysis utilizing active subnetworks (An active subnetwork can be defined as a group of interconnected genes in a protein-protein interaction network (PIN) that predominantly consists of significantly altered genes). It identifies gene sets that form active subnetworks in a protein-protein interaction network using a list of genes provided by the user. It then performs pathway enrichment analyses on the identified gene sets. In most enrichment approaches, relational information captured in the graph structure of a PIN is overlooked as genes in the network neighborhood of significant genes are not taken into account. The approach pathfindR uses for exploiting interaction information to enhance pathway enrichment analysis is active subnetwork search. Briefly, active subnetwork search enables inclusion of genes that are not significant genes themselves but connect significant genes. This results in the identification of phenotype-associated connected significant subnetworks. Initially identifying active subnetworks in a list of significant genes and then performing pathway enrichment analysis of these active subnetworks efficiently, pathfindR exploits interaction information between the genes. This, in turn, helps pathfindR uncover relevant mechanisms underlying the disease.

Support Vector Machines-Recursive Cluster Elimination (SVM-RCE) is a machine learning algorithm based on grouping/clustering gene expressions for scoring each cluster of genes (Yousef et al., 2007). Interest in this approach has grown over time and a number of publications based on SVM-RCE that have successfully applied this approach to identifying those features directly associated with a disease/condition are being published. This increasing interest is based on a reconsideration of how feature selection in biological datasets can benefit from considering the biomedical relationships of the features in the selection process. The usefulness of SVM-RCE then led to the development of additional computational tools. Similar studies for SVM-RCE were carried out (Harris & Niekerk, 2018; Lazzarini & Bacardit, 2017) indicating the importance of the merit of SVM-RCE approach. The study of Deshpande et al. (2010) is a derivative of SVM-RCE algorithm with small modification for disease state prediction. Additionally, they have used our invented term “recursive cluster elimination”.

Most interestingly, the study of Zhao, Wang & Chen (2017) has used the SVM-RCE tool for comparison tasks applied for detection on expression profiles for identifying microRNAs related to venous metastasis in hepatocellular carcinoma.

SVM-RNE (Yousef et al., 2009) is a similar approach to SVM-RCE, and uses the GXNA (Nacu et al., 2007) tool to extract the genes networks from the gene expression data. Those networks serve as groups/clusters of genes that are subject to the rank procedure. A similar study to SVM-RNE was carried out by Johannes et al. (2010) for the integration of pathway knowledge into a reweighted recursive feature elimination approach for risk stratification of cancer patients.

The term knowledge-driven variable selection (KDVS) is a similar term of integration of biological knowledge in the process of feature selection. In Zycinski et al. (2013), the authors proposed a KDVS framework, which uses a priori biological knowledge in high–throughput data analysis, and applied this framework to SVM-RNE.

The most recent tool that integrates biological knowledge for grouping the genes was maTE (Yousef, Abdallah & Allmer, 2019), which uses the same approach based on the interactions of microRNAs (miRNA) and their gene targets. The maTE approach is different from SVM-RCE and SVM-RNE in that it integrates additional input to the algorithm which is the information about miRNA and its target set.

The benefit of integration of biological knowledge led us to suggest a new tool called CogNet, that integrates biological knowledge derived from integrating the pathfindR tool into an integrative approach. In CogNet, the pathfindR tool serves as the biological grouping function allowing the main algorithm to rank active-subnetwork-oriented KEGG pathway enrichment analysis results. The details of the tool will be described in the following sections.

## Materials and Methods

**Figure 1:**
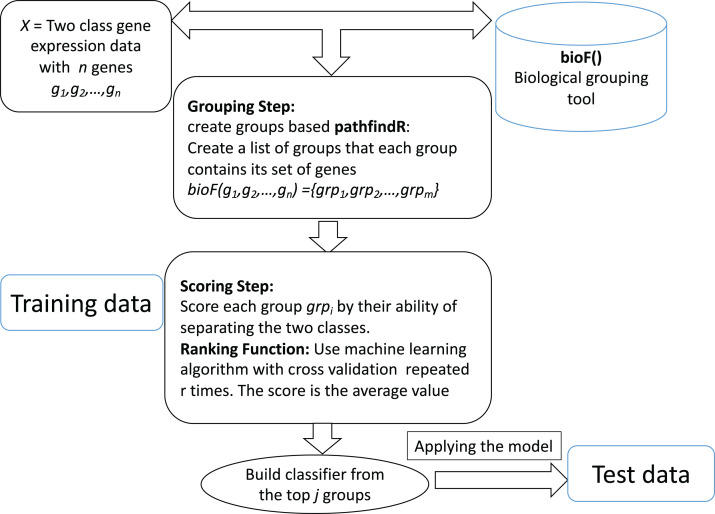
General workflow for integrating biological information for grouping the genes by *bioF()* function. *BioF()* could be microRNA targets association, KEGG pathway association or other association.

The computational tool CogNet that we developed is based on the concept of integration of biological knowledge with machine learning in order to perform two tasks: the first task is ranking the groups of genes (in this case, pathway genes) and then use the top groups (significant groups) to build a machine learning model. Figure 1 displays the general workflow of integrating biological information for grouping the genes by a biological grouping functions. The CogNet components meet the general approach of the integration of biological knowledge with machine learning as described in Fig. 1. In fact, the tools SVM-RCE, SVM-RNE, and maTE also fit the general approach described in Fig. 1.

Let us assume that we are given a two-class data *D*, which consists of *k* samples and *n* genes. Let us assume that the biological grouping function groups the *n* genes into *m* groups as following: *bioF(g*_*1*_*,g*_*2*_*,…,g*_*n*_*) = {grp*_*1*_*,grp*_*2*_*,…,grp*_*m*_*}. bioF()* can also assign some genes to a specific group that contains genes about which there is no biological knowledge.

The *bioF()* function is used in order to group/cluster the genes using biological information that could be associated with a specific biological concept. For example, *bioF()* could group genes according to their miRNA targets (as the tool maTE did), or might be according to disease, meaning that groups of genes are associated with specific diseases.

The ranking step is based on the machine learning algorithm used. In order to estimate the significance of each group *grp*_*i*_ of genes, the following algorithm is applied:

1. Create a new data D* that contains only genes from the *grp*_*i*_.
2. Apply cross validation using the ML algorithm.
3. Assign a score to *grp*_*i*_. The score is the average of the performance metric (could be accuracy, the area under the curve, f-measure, etc.)

### pathfindR

In this section, we describe the pathfindR tool that serves as the *bioF()* function (Fig. 1) in the CogNet tool.

Active-subnetwork-oriented KEGG pathway enrichment analysis of the proteins was conducted using the R package pathfindR (Ulgen, Ozisik & Sezerman, 2019). Active subnetworks are subnetworks within the PIN (BioGRID, by default) that have a locally maximal score (based on the provided significance values). Active subnetworks define distinct disease-associated sets of interacting genes, whether discovered through the original analysis or discovered because of being in interaction with a significant gene.

**Figure 2:**
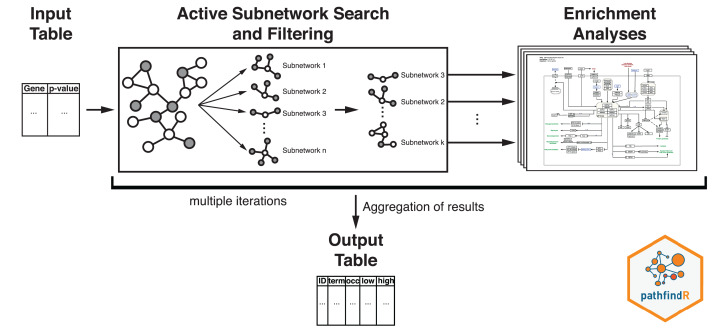
Flow diagram of the pathfindR active-subnetwork-oriented enrichment analysis approach. Image credit: Coort S, Hanspers K, Waagmeester A, Defay A et al. (https://www.wikipathways.org/index.php/Pathway:WP1403), CC0 1.0 Universal (CC0 1.0).

The workflow of pathfindR is presented in Fig. 2. After processing the input (to filter the differential expression results, the *p* value threshold was chosen as 0.2), pathfindR maps the input genes onto a PIN. Using the mapped genes, an active subnetwork search (with the greedy approach as default) is performed. The resulting active subnetworks are then filtered based on their scores and the number of significant genes they contain. This filtered list of active subnetworks is then used for enrichment analyses (over-representation analysis via hypergeometric-distribution-based tests), that is, using the genes in each of the active subnetworks, the significantly enriched pathways are identified. Enriched pathways with adjusted *p* values larger than the given threshold (0.2 was used) are discarded and the lowest adjusted *p*-value (overall active subnetworks) for each term is kept. This process of “active subnetwork search + enrichment analyses” is repeated for a selected number of iterations (default is 10), performed in parallel. Over all iterations, the lowest and the highest adjusted-p values, as well as the number of occurrences over all iterations are reported for each significantly enriched pathway.

### The CogNet tool

**Figure 3:**
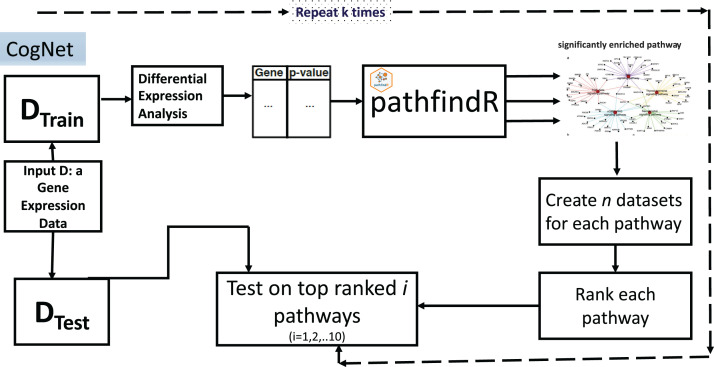
CogNet workflow.

The CogNet algorithm is based on the general approach of integrating biological information for grouping the genes as described in Fig. 1. Overall, the tool is composed of two components. The first component is the pathfindR step that is serving to generate the groups of genes, which are enriched KEGG pathways. Then, the second component is applied to rank those groups in terms of their contribution to separate the two-class data *D*. The workflow of the tool is presented in Fig. 3.

The CogNet starts by splitting the data into two parts. The first part is the training part that is used in order to rank the pathways produced by the tool pathfindR. The test part is utilized at the end of the algorithm in order to estimate the performance of the CogNet.

A list of genes and their *p*-values represented in a table is created to serve as input to pathfindR (Table 1). The list is computed using Student’s *t*-test to assign for each gene its differential expression significance (*p*-value). The pathfindR tool is invoked to create *n* significant KEGG pathways, from now on, we refer to this as *n* groups of genes *grp*_*1*_*,grp*_*2*_*,…,grp*_*n*_. As a preprocess to the step of the ranking, each KEGG pathway is producing one data set out of the final data *D* that containing only genes of the specific pathway. Thus, *n* datasets D*_i_ i = 1,2,…n, (with |grp*_i_*| as the number of genes) are created to be subject for the Rank step. Table 2 presents an example input table to Rank step.

**Table 1:** Example of input table to the tool pathfindR.

Gene	*p*-value
SCARNA15	0.015915
CACNA1D	0.015915
IP6K3	0.015915
MIR1304	0.015915
DLX2	0.015915
SNORD77	0.015915
SGCG	0.015915
TCF21	0.015915
RPS21	0.021748

**Note:**

The table consist from list of genes and its *p*-values.

**Table 2:** Example of input table to the Rank step.

ID	*p*-value	PathWay Genes
hsa05165	8.79E−06	FZD7, THBS4, COL4A1, COL9A2, TNC, ITGA8, PTK2, PPP2CA, CCND2, IKBKB, ATP6V1G2, ATP6V0A2, JAG1, MAML3
hsa04151	1.40E−05	FGF2, IGF1, FLT3LG, ANGPT1, COL4A1, COL9A2, THBS4, TNC, ITGA8, PTK2, LPAR1, GNB1, MLST8, RPS6, PPP2CA, CCND2, IKBKB
hsa04062	2.14E−05	CCL18, ADCY4, ADCY5, IKBKB, ROCK2, GNB1, PTK2, PRKCB, GRK5
hsa03010	1.82E−04	RPS6, RPS12, RPS21, RPS29, RPL17-C18orf32, RPL18, RPL22L1, RPL31
hsa05170	4.60E−05	PTK2, PRKCB, GNB1, IKBKB, CCNB3

**Note:**

The list consists from three columns, ID is the KEGG Pathway id, *p*-value is the *p*-value of the pathways computed by pathfidR while the last column is the list of genes belonging to the KEGG pathway.

The Rank step is computed as a Monte Carlo cross-validation (MCCV), where for each D*_i_ a score is assigned as the mean of computing accuracy of *r* iteration of splitting the data into two parts one for training and second for testing (for example, 80% training and 20% testing) applying random forest (or another ML algorithm such as SVM). The output of the stage is a sorted list of KEGG pathways according to the score assigned in the Rank step. Let refer to this list as grp*_1_, grp*_2_, …, grp*_n_. Table 3 presents an example of the output of Rank step.

**Table 3:** Example output of Rank step.

ID	Acc	Sen	Spe	Rec	Prec	Fm
hsa05165	1.00	1.00	1.00	1.00	1.00	1.00
hsa04151	0.93	0.90	1.00	0.90	1.00	0.93
hsa03010	0.87	0.80	1.00	0.80	1.00	1.00
hsa05170	0.93	0.90	1.00	0.90	1.00	0.93
hsa05163	0.87	1.00	0.60	1.00	0.87	0.92
hsa04530	1.00	1.00	1.00	1.00	1.00	1.00
hsa04064	0.87	1.00	0.60	1.00	0.87	0.92
hsa05418	0.73	0.70	0.80	0.70	0.92	0.87
hsa05200	1.00	1.00	1.00	1.00	1.00	1.00
hsa04110	1.00	1.00	1.00	1.00	1.00	1.00

**Note:**

The values are the mean of each performance metric appearing on the column title. Acc, accuracy; Sen, sensitivity; Spe, specificity; Rec, recall; Prec, precession; Fm, F-measure.

The final step is to compute the performance of the CogNet by training the RF on top pathways and test on the main test data (from the first stage of the algorithm).

- *gene_list*={}
- for *i* = 1 to *n*:
  - *gene_list* = gene_list ∪ grp*_n_
  - create Tr to be the data D_Train_ with just genes belongs to *gene_list*.
  - train RF on Tr and create an RF model.
  - create Tst to be the data D_Test_ with just genes belongs to gene_list.
  - *res{i}* = performance of applying RF model on Tst.

One of the outputs of the CogNet is a table that reports the performance on top 1, 2,…, *n*. However, we have created a table only for the top 10. We should state that *n* is a variable and is dependent on the data. Sometimes, *n* may not reach 10 because pathfindR reports only the significant KEGG pathways. In the worst case, the output of pathfindR could be an empty list.

Another output is the significant list of KEGG pathways and also the significant genes.

We run the CogNet algorithm multiple times (*k* times) where each time the data is split into 90% for training and 10% for testing. The CogNet keeps track of each KEGG pathway and the number of times appears on top of the list. Additionally, CogNet keeps track of the genes that belong to the pathway and how many times they were on the top. An example of input of top pathways and genes produced by CogNet is presented in Table 4.

**Table 4:** Example of output of top pathways and top genes.

KEGG Pathway	count	Genes
hsa03460	77	BRIP, RMI2,UBE2T
hsa05034	70	CREB3L4,GNB4,HIST1H2AK,HIST1H2BC,HIST1H2BJ,HIST1H4H,HIST2H2AA4,PPP1R1B
hsa05322	53	COL4A3,HIST1H2AK,HIST1H2BC,HIST1H2BJ,HIST1H4H,HIST2H2AA4
hsa04151	50	COL1A1,PRLR

### Implementation

We have decided to use the free and open-source platform Knime (Berthold et al., 2009) due to its simplicity and very useful graphical representations. Additionally, Knime is a highly integrative tool. A script for performing analysis with pathfindR was imported as an R node to the Knime workflow.

The Knime workflow consists mainly of nodes where each node has its own functionality. Meta- node is created as a collection of nodes that has a specific task to perform.

**Figure 4:**
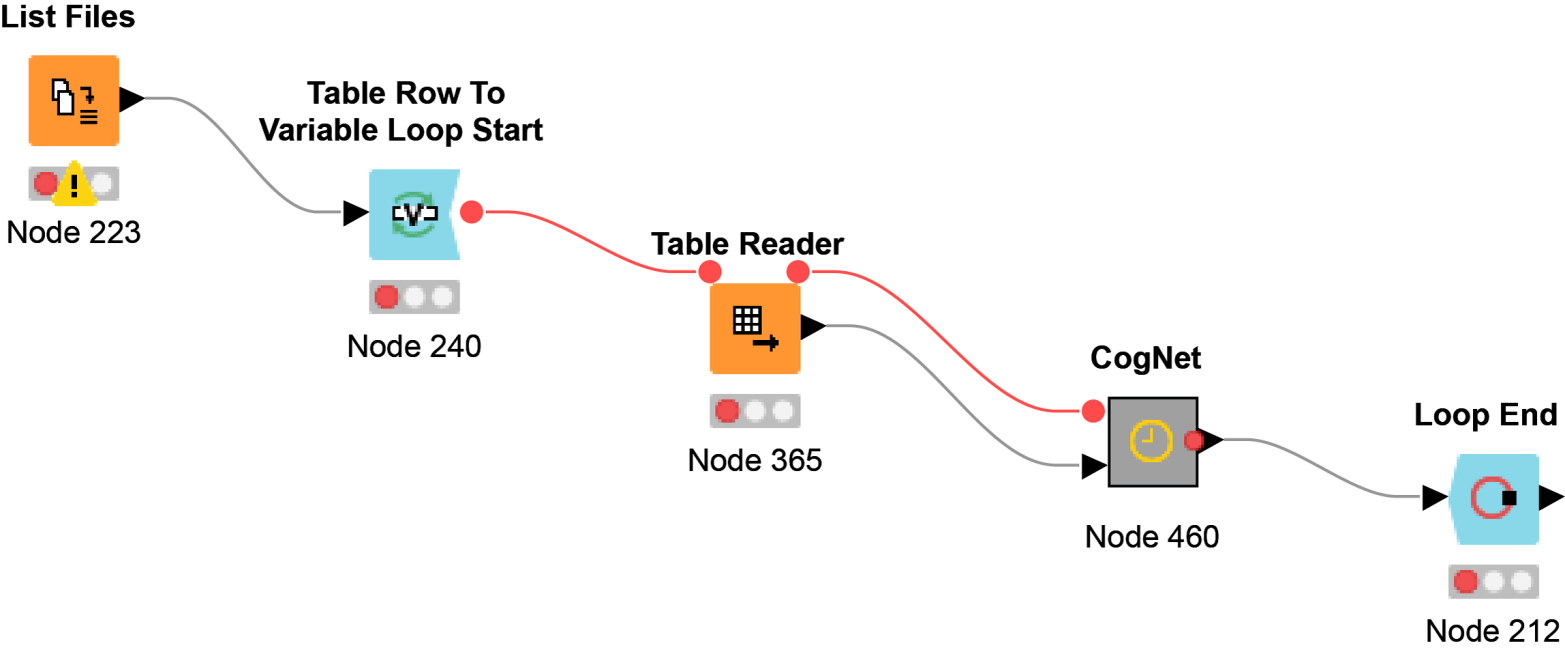
Multi-File CogNet workflow that applied on multiple datasets.

The workflow Multi-File CogNet is presented in Fig. 4. It starts by uploading a list of the names of the datasets (URL) by the “List Files node”. Then a loop over those datasets to read each data by the node “Table Reader” and send it as input to the CogNet tool (CogNet meta-node).

**Figure 5:**
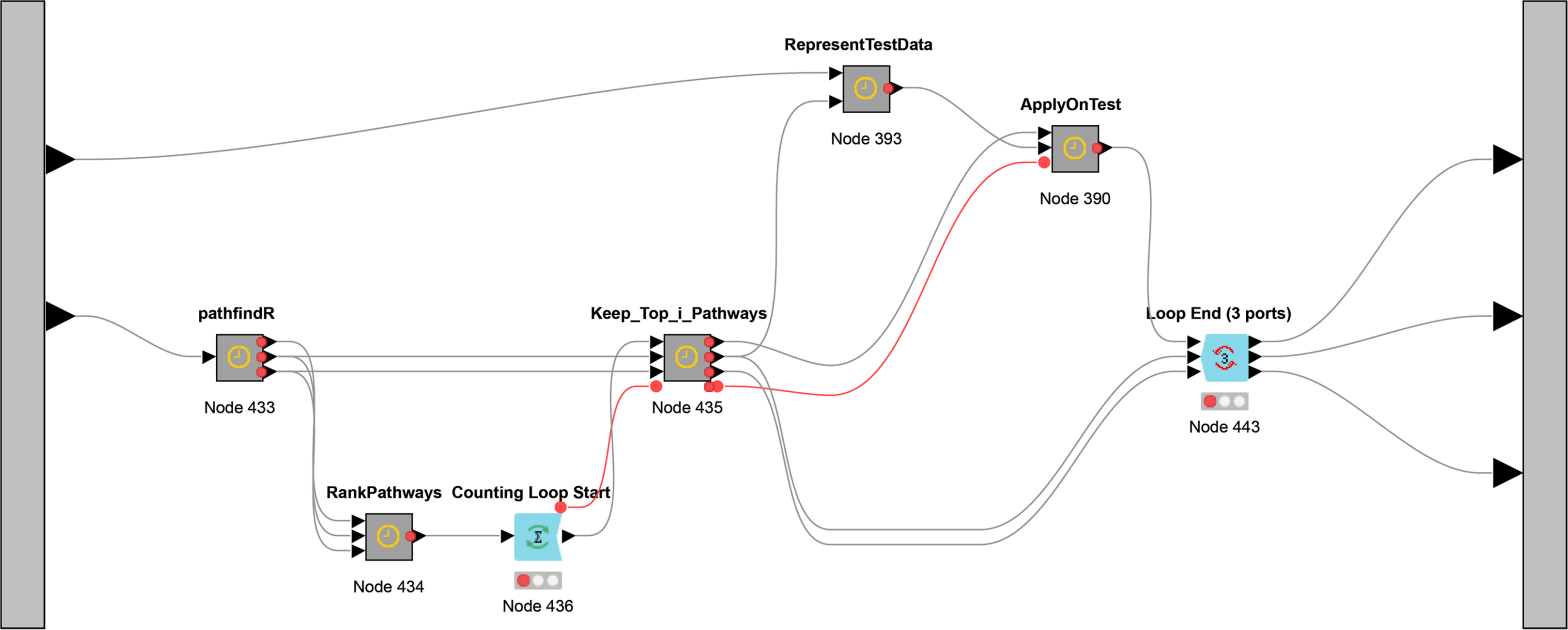
The main workflow (as meta-nodes) for CogNet.

While Fig. 3 presents the flowchart of CogNet algorithm, Fig. 5 presents the implementation of CogNet as a Knime workflow with its meta-nodes. The input has two ports, where the first port is for the test data, the second port is for the training data. The training data is passed to the tool pathfindR (one of the meta-nodes) to process the data and get as an output a sorted table of significant pathways. The “RankPathWays” meta nodes perform the task of the ranking, while the flow between “Counting Loop start” and “Loop End” is for performing testing on top *i* pathways. It ranges from 1 to 10.

Additional task for the node “Loop End” is to collect all the results and send them out to be processed and save the results.

### Gene expression data

A total of 13 human gene expression datasets were downloaded from the gene expression omnibus (Clough & Barrett, 2016) at NCBI. For all datasets disease (positive) and control (negative) data were available (Table 5). Those 13 datasets served to test the CogNet and for comparison with other two tools maTE and SVM-RCE.

**Table 5:** Description of the 10 data sets used in our study. The data sets are obtained from GEO. Each entry has the GEO code the name of the data, the number of samples and the classes of the data.

GEO Accession	Title	Sample count	Classes
GDS1962	Glioma-derived stem cell factor effect on angiogenesis in the brain	180 pos = 157 neg = 23	non-tumor = 23 (neg) astrocytomas = 26 (pos) glioblastomas = 131 (pos)
GDS2519	Early-stage Parkinson’s disease: whole blood	105 pos = 50 neg = 55	healthy control = 22 (neg) neurodegenerative disease control = 33 (neg) Parkinson disease = 50 (pos)
GDS3268	Colon epithelial biopsies of ulcerative colitis patients	202 pos = 73 neg = 129	normal = 73 ulcerative colitis = 129
GDS2547	Metastatic prostate cancer (HG-U95C)	164 pos = 75 neg = 89	normal = 75 tumor = 89
GDS5499	Pulmonary hypertensions: PBMCs	140 pos = 99 neg = 41	control = 41 (neg) idiopathic pulmonary arterial hypertension = 30 (pos) scleroderma-associated pulm. arterial hypert. = 42 (pos) systemic sclerosis (SSc) without pulm. hypert. = 19 (pos) SSc, interstitial lung disease & pulm. hypert. = 8 (pos)
GDS3646	Celiac disease: primary leukocytes	132 pos = 110 neg = 22	healthy control =22 celiac disease = 110
GDS3874	Diabetic children: peripheral blood mononuclear cells (U133A)	117 neg = 24 pos = 93	healthy = 24 type 1,2 diabetes =93
GDS3837	Non-small cell lung carcinoma in female nonsmokers	120 pos = 60 neg = 60	Lung Cancer = 60 Control = 60
GDS5037	Severe asthma: bronchial epithelial cell	108 Pos = 88 Neg = 20	mild asthma = 50 control = 20 severe asthma = 38
GDS4516_4718	Colorectal cancer: laser microdissected tumor tissues Colorectal cancer: homogenized tumor tissues	148 pos = 104 neg = 44	laser microdissected tumor tissues = 104 homogenized tumor tissues = 44
GSE4107 (GDS2609)	Colonic mucosa	22 pos = 12 neg = 10	colonic mucosa of healthy control = 12 colonic mucosa patients = 10
GSE15573 (GDS3794)	Rheumatoid Arthritis (RA) Patients	33 pos = 18 neg = 15	18 Rheumatoid Arthritis (RA) 15 Control
GSE5594 (GDS4824)	Prostate cancer Analysis of malignant and benign prostate tissues	21 pos = 13 neg = 8	prostate cancer = 13 normal = 8

### Model performance evaluation

For each established model, we calculated a number of statistical measures like sensitivity, specificity, and accuracy to evaluate model performance. The following formulations were used to calculate the statistics (with TP: true positive, FP: false positive, TN: true negative and FN referring to false negative classifications):

Sensitivity (SE, Recall) = TP/(TP + FN)

Specificity (SP) = TN/(TN + FP)

Accuracy (ACC) = (TP + TN)/(TP + TN + FP + FN); ACC

Additionally, the Area Under the Receiver Operating Characteristic (ROC) Curve measure (AUC) (Bradley, 1997) is an estimate of the probability that a classifier will rank a randomly chosen positive instance higher than a randomly chosen negative instance.

All reported performance measures refer to the average of 10-fold MCCV.

Some of the data sets used by the classifier are imbalanced, which can influence the classifier to the advantage of the set with more samples. This is known as the problem of the imbalanced class distribution. We have applied an under-sampling approach that reduces the number of samples of the majority class to the minority class, thus reducing the bias in the size distribution of the data subsets. We choose to apply the under-sampling of ratio 1:2.

### Availability and implementation

The Knime workflow, implementing CogNet, is available at https://malikyousef.com -> Bioinformatics Tools and GitHub at https://github.com/malikyousef/miRcorrNet. The DOI of the tool is https://doi.org/10.5281/zenodo.4273942.

## Results

We have considered 13 gene expression data sets to test CogNet and for comparison with other similar tools. To our knowledge, no tools similar to CogNet exists. Nonetheless, we compare CogNet with tools that have similar merit of grouping and rankings, maTE and SVM-RCE. Although the purpose of the comparison is not to prove a higher performance, it outperforms maTE and gets similar performance to SVM-RCE with advantage of using a very smaller number of genes than SVM-RCE. Additionally, we have run CogNet with two versions, where the CogNet_SVM uses the SVM for the scoring and classification while the CogNet_RF uses Random Forest (RF) for Scoring and classification. The results for CogNet-SVM is provided in the Supplemental Data.

For each tool other than SVM-RCE, we have obtained the performance over the top 1–10 groups that were ranked by the scoring stage. For SVM-RCE we obtained the performance starting by 1,000 genes and 100 clusters, then reduced 10% at each iteration. For comparing purposes, we consider the last 10 clusters. Table 6 presents in detail all the results obtained applying the CogNet_RF tool. The AUC measure is presented. The columns #G present the average of the number of genes over the 10 iterations was applied as a cross-validation.

**Table 6:** (A–C) A summary result table presenting the AUC of each tool over 13 data sets. #Clusters/#Groups are related to the number of clusters for SVM-RCE and groups for maTE and CogNet. AUC is average of for the performance of area under curve while #G is the average number of genes for each level.

#Clusters/#Groups	SVM-RCE												
	GDS1962	GDS2519	GDS2547	GDS3268	GDS3646	GDS3837	GDS3874	GDS4516_4718	GDS5037	GDS5499	GDS2609	GDS3794	GDS4824
	AUC	AUC	AUC	AUC	AUC	AUC	AUC	AUC	AUC	AUC	AUC	AUC	AUC
10	1.00	0.50	0.84	0.90	0.90	0.97	0.75	1.00	0.48	0.95	1.00	1.00	1.00
9	1.00	0.49	0.84	0.90	0.82	0.98	0.81	1.00	0.57	0.93	0.99	0.98	1.00
8	1.00	0.49	0.85	0.90	0.87	0.98	0.79	1.00	0.53	0.94	0.96	0.98	1.00
7	1.00	0.49	0.85	0.89	0.86	0.98	0.78	1.00	0.53	0.93	0.97	0.98	1.00
6	1.00	0.48	0.85	0.89	0.87	0.98	0.81	1.00	0.53	0.95	0.96	0.98	1.00
5	1.00	0.47	0.85	0.88	0.87	0.98	0.79	1.00	0.50	0.94	0.96	0.98	1.00
4	1.00	0.44	0.84	0.88	0.87	0.98	0.78	1.00	0.44	0.95	0.96	0.98	1.00
3	1.00	0.46	0.84	0.89	0.87	0.98	0.74	1.00	0.48	0.95	0.93	0.98	1.00
2	1.00	0.44	0.81	0.87	0.84	0.98	0.79	1.00	0.43	0.93	0.91	0.98	1.00
1	1.00	0.45	0.80	0.84	0.76	0.97	0.66	1.00	0.50	0.90	0.90	0.95	1.00
Avg	1.00	0.47	0.84	0.88	0.85	0.98	0.77	1.00	0.50	0.94	0.95	0.98	1.00
#Clusters/#Groups	#G	#G	#G	#G	#G	#G	#G	#G	#G	#G	#G	#G	#G
10	845	801	896	852	341	904	569	734	505	507	676	442	611
9	827	791	875	818	311	868	586	712	492	493	652	437	536
8	806	752	804	761	272	807	572	675	483	460	638	421	515
7	760	715	747	702	245	772	542	633	436	437	625	413	491
6	720	678	686	644	224	694	499	580	428	394	584	397	468
5	678	638	636	595	209	604	464	555	399	329	558	361	453
4	593	575	544	568	169	486	432	444	348	265	511	340	422
3	429	504	466	470	142	419	375	319	307	225	470	310	381
2	363	444	449	377	74	283	318	188	238	171	390	227	310
1	237	266	373	211	47	137	224	91	131	62	264	120	211

#Clusters/#Groups	CogNet_RF												
	GDS1962	GDS2519	GDS2547	GDS3268	GDS3646	GDS3837	GDS3874	GDS4516_4718	GDS5037	GDS5499	GDS2609	GDS3794	GDS4824
	AUC	AUC	AUC	AUC	AUC	AUC	AUC	AUC	AUC	AUC	AUC	AUC	AUC
10	1.00	0.65	0.84	0.72	0.76	0.98	0.90	1.00	0.68	0.95	1.00	0.93	0.92
9	0.99	0.65	0.85	0.76	0.76	0.97	0.86	1.00	0.66	0.94	1.00	0.95	1.00
8	0.99	0.63	0.86	0.75	0.76	0.99	0.87	1.00	0.64	0.93	1.00	0.98	1.00
7	0.99	0.68	0.86	0.74	0.76	0.98	0.87	1.00	0.70	0.93	1.00	1.00	1.00
6	0.98	0.55	0.84	0.74	0.68	0.98	0.85	1.00	0.71	0.93	1.00	1.00	1.00
5	0.99	0.74	0.83	0.75	0.68	0.98	0.89	1.00	0.72	0.94	1.00	1.00	0.80
4	0.98	0.68	0.83	0.72	0.66	0.97	0.89	1.00	0.69	0.94	1.00	0.93	0.80
3	0.98	0.66	0.83	0.73	0.68	0.96	0.91	1.00	0.71	0.94	1.00	0.88	0.90
2	0.98	0.65	0.78	0.73	0.64	0.97	0.89	1.00	0.72	0.93	1.00	0.85	0.90
1	0.99	0.59	0.74	0.75	0.72	0.91	0.86	1.00	0.74	0.93	1.00	0.88	0.85
Avg	0.99	0.65	0.83	0.74	0.71	0.97	0.88	1.00	0.70	0.94	1.00	0.94	0.92
#Clusters/#Groups	#G	#G	#G	#G	#G	#G	#G	#G	#G	#G	#G	#G	#G
10	123	65	62	65	15	34	23	45	16	52	95	27	51
9	110	56	55	59	14	33	22	41	15	50	84	26	45
8	98	53	50	53	13	31	22	39	14	48	77	23	35
7	88	42	42	47	13	29	19	35	14	46	68	22	26
6	80	34	36	41	11	26	19	30	13	44	62	20	26
5	70	26	30	35	10	24	17	26	9	41	52	18	22
4	62	27	25	28	8	20	16	22	7	37	41	15	17
3	52	22	19	21	7	17	13	17	6	29	32	13	13
2	38	16	13	13	6	13	10	13	3	21	22	9	9
1	8	8	7	6	5	6	6	8	4	8	11	5	5

**Figure 6:**
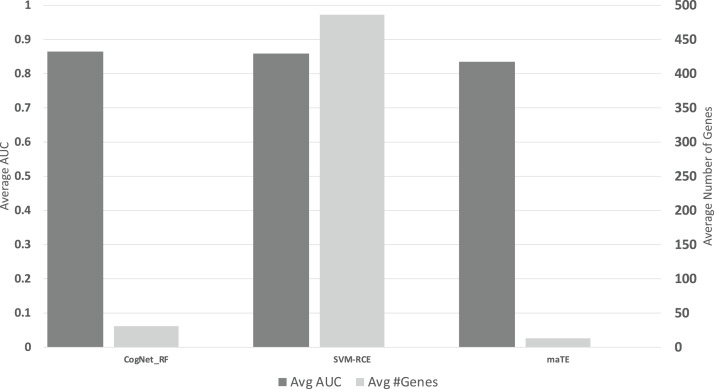
The average of the results for the three tools. The upper part is the performance AUC measurement while the lower part is the number of genes (#G).

Figure 6 presents the average of all AUC results over the 13 datasets on the 10 clusters/groups, for each tool (Avg AUC bar), while the Avg#Genes bar represent the average of the number of genes over the 13 datasets on the 10 clusters/groups.

The results presented in Tables 6A–6C and Fig. 6, indicate that on average CogNet outperforms maTE by 3% while getting similar results with SVM-RCE. Considering the number of genes, SVM-RCE is 37 folds greater than CogNet. Additional observation that both tools SVM-RCE and maTE failed to reach reasonable results on the data GDS2519, while CogNet reached performance of about 65–70%.

### Validation of the results

We have conducted further analysis using the datasets (GSE15573, GSE4107 and GSE55945) that was considered in the pathfindR study. GSE15573 consists of 33 samples corresponding to 18 Rheumatoid Arthritis (RA) Patients and 15 Controls. The aim of this study is to identify peripheral blood gene expression profiles for RA patients. The study considers the standard statistical approaches in order to detect the significant genes by ANOVA with False Discovery Rate (FDR < 5%). The Gene Ontology (GO) in the PANTHER database is applied to identify biological processes.

GSE4107 consists of 10 colonic mucosa of healthy control samples and 12 patient samples. Patients and controls were age- (50 or less), ethnicity- (Chinese) and tissue-matched. The analysis to detect significant genes was based on *T*-test, hierarchical clustering, mean fold-change and principal component.

GSE55945 consists of 13 prostate cancer samples and 8 normal. The aim of the study is to compare the expression levels between malignant and benign prostate tissues.

We have run the CogNet tool on those three datasets obtaining the performance on top significant pathways. The AUC values are presented in Table 6.

### Association of the top pathways with the disease under study

For each dataset, the top 10 groups (pathways) identified by CogNet were manually examined in the literature for a possible association with the disease under study. Literature support for the top 10 pathways per each dataset are presented in Table 7.

**Table 7:** Association of the top pathways with the disease under study. “Dataset” indicates the dataset GEO ID.

Dataset	Investigated disease	ID	Pathway	Literature support	PMID
GSE15573	Rheumatoid Arthritis	hsa04932	Non-alcoholic fatty liver disease (NAFLD)	None	None
GSE15574	Rheumatoid Arthritis	hsa04120	Ubiquitin mediated proteolysis	Aberration of this system leads to the dysregulation of cellular homeostasis and the development of multiple inflammatory and autoimmune diseases, including rheumatoid arthritis.	16978533
GSE15575	Rheumatoid Arthritis	hsa05016	Huntington disease	None	None
GSE15576	Rheumatoid Arthritis	hsa04714	Thermogenesis	None	None
GSE15577	Rheumatoid Arthritis	hsa04723	Retrograde endocannabinoid signaling	Endocannabinoids, a group of endogenous bioactive lipids, have immunomodulatory effects able to influence both inflammation and pain in rheumatic disease, including rheumatoid arthritis.	29164003, 28857069
GSE15578	Rheumatoid Arthritis	hsa05010	Alzheimer disease	None	None
GSE15579	Rheumatoid Arthritis	hsa04140	Autophagy	Deregulation of autophagic pathway has recently been implicated in the pathogenesis of several autoimmune diseases, including rheumatoid arthritis.	30072986
GSE15580	Rheumatoid Arthritis	hsa04621	NOD-like receptor signaling pathway	NOD-like receptors are being implicated in the pathology of RA and other rheumatic diseases.	19835640
GSE15581	Rheumatoid Arthritis	hsa05012	Parkinson disease	None	None
GSE15582	Rheumatoid Arthritis	hsa05203	Viral carcinogenesis	None	None
GSE4107	Colorectal Cancer	hsa04915	Estrogen signaling pathway	None	None
GSE4107	Colorectal Cancer	hsa04662	B cell receptor signaling pathway	None	None
GSE4107	Colorectal Cancer	hsa04012	ErbB signaling pathway	ERBB pathway may have a role in both normal colon epithelial cell differentiation and malignant transformation.	27270421
GSE4107	Colorectal Cancer	hsa05140	Leishmaniasis	None	None
GSE4107	Colorectal Cancer	hsa05224	Breast cancer	None	None
GSE4107	Colorectal Cancer	hsa04510	Focal adhesion	Cancer cells exhibit highly altered focal adhesion dynamics.	28476046
GSE4107	Colorectal Cancer	hsa04210	Apoptosis	Abnormalities in apoptotic function contribute to both the pathogenesis of colorectal cancer and its resistance to chemotherapeutic drugs and radiotherapy.	15479695
GSE4107	Colorectal Cancer	hsa04010	MAPK signaling pathway	MAPK signaling plays an important part in progression of colorectal cancer.	15863380
GSE4107	Colorectal Cancer	hsa05166	Human T-cell leukemia virus 1 infection	None	None
GSE4107	Colorectal Cancer	hsa05200	Pathways in cancer	"Meta"-pathway of cancer pathways.	None
GSE55945	Prostate Cancer	hsa04110	Cell cycle	Dysregulation of the cell cycle is implicated in the biology of many cancers, including PCa.	7997877, 9096291, 18301781
GSE55945	Prostate Cancer	hsa04360	Axon guidance	None	None
GSE55945	Prostate Cancer	hsa04390	Hippo signaling pathway	The hippo pathway effector YAP regulates motility, invasion, and castration-resistant growth of prostate cancer cells.	25645929
GSE55945	Prostate Cancer	hsa05166	Human T-cell leukemia virus 1 infection	None	None
GSE55945	Prostate Cancer	hsa04014	Ras signaling pathway	Ras signaling plays an important role in prostate cancer progression and is a possibly mediator of hormone resistance.	14689577, 20718703
GSE55945	Prostate Cancer	hsa01040	Biosynthesis of unsaturated fatty acids	Alterations in lipid metabolism, and specifically the uptake and synthesis of fatty acids (FAs), comprise a well-documented aspect of metabolic reprograming in cancer.	31598388
GSE55945	Prostate Cancer	hsa05163	Human cytomegalovirus infection	None	None
GSE55945	Prostate Cancer	hsa04350	TGF-beta signaling pathway	TGF-beta signaling has pivotal roles in tumorigenesis and tumor progression	26774024, 29115550
GSE55945	Prostate Cancer	hsa04010	MAPK signaling pathway	MAPK signaling pathways act through their effects on apoptosis, survival, metastatic potential, and androgen-independent growth in prostate cancer	22046506
GSE55945	Prostate Cancer	hsa04933	AGE-RAGE signaling pathway in diabetic complications	None	None

**Note:**

“Investigated Disease” indicates the disease investigated by the study. “ID” and “Pathway” indicate the KEGG ID and pathway name of the top pathway, respectively. “Literature support” provides a brief summary of literature support for the pathway-disease association. “PMID” indicates the PubMed ID(s) of the supporting study/studies.

For GSE15573, the rheumatoid arthritis dataset, 4 out the top 10 pathways were found to be supported by literature to be associated with rheumatoid arthritis biology. For GSE4107, comparing colorectal cancer patients to healthy controls, 5 out of the top 10 pathways were supported by literature to be associated with colorectal cancer. Finally, for GSE55945, the dataset comparing human prostate benign and malignant tissue, 6 out of the top 10 pathways were found to be associated with prostate cancer.

The CogNet results highlighted several new pathways that could contribute to the identification of innovative clinical biomarkers for diagnostic procedures and therapeutic intervention.

## Discussion and conclusions

We have presented a novel tool called CogNet that is based on ranking and classification and integration of biological knowledge. CogNet is developed on top of the tool pathfindR to add to its functionality the ability to perform classification using the enriched KEGG pathways as features. CogNet outputs to the user the performance and a list of significant KEGG pathways that we believe will contribute to a better and deep understanding of the data under investigation.

There is similarity between CogNet and maTE in that both rank a group of genes for classification tasks. However, CogNet is using the biological information of the KEGG pathways that was processed by an enrichment procedure to suggest a list of significant pathways, then CogNet ranks those pathways in terms of their contribution for separating the two-class of the given data (classification task). However, maTE uses prior information about microRNA and its target genes to group the genes. This grouping is not related to the expression of the genes as CogNet is considering. Moreover, SVM-RCE is using the clustering algorithm k-means in order to group the genes into clusters, that means that groups are related to the expression of the genes.

As a future work, we would develop CogNet to explore the effectiveness of different combinations of the KEGG pathways in the data, that means instead of ranking each pathway’s genes individually, we will use a different approach to rank those groups simultaneously. In the current version, CogNet ranks each KEGG pathway individually by performing internal cross validation.

Additionally, we are working to integrate CogNet and maTE where the process of the rank will be applied on the groups generated by the biological functions used in each tool. As a result, the discovery of the significant pathways and microRNA targets genes will suggest to the biology researcher to explore the role of pathways and microRNA together in the same data.

The success of CogNet, maTE and SVM-RCE is suggesting that more computational approaches need to be developed based on the merit of integration of biological information into the machine learning algorithm.

## Additional Information and Declarations

### Data Availability

The following information was supplied regarding data availability: Data is available at NCBI GEO: GDS1962, GDS2519, GDS3268, GDS2547, GDS5499, GDS3646, GDS3874, GDS3837, GDS5037, GDS4516, GDS4517, GDS4718, GSE4107, GDS2609, GSE15573, GDS3794, GSE5594, GDS4824. The code is available at GitHub: https://github.com/malikyousef/miRcorrNet. The DOI of the tool is available at Zenodo: malikyousef. (2020, November 14). malikyousef/miRcorrNet: miRcorrNet (Version v1.0). Zenodo. DOI 10.5281/zenodo.4273942.
